# Reduced graphene oxide-TiO_2_ nanocomposite as a promising visible-light-active photocatalyst for the conversion of carbon dioxide

**DOI:** 10.1186/1556-276X-8-465

**Published:** 2013-11-06

**Authors:** Lling-Lling Tan, Wee-Jun Ong, Siang-Piao Chai, Abdul Rahman Mohamed

**Affiliations:** 1 Low Carbon Economy (LCE) Group, Chemical Engineering Discipline, School of Engineering, Monash University, Jalan Lagoon Selatan, Bandar Sunway, Selangor 46150, Malaysia; 2 Low Carbon Economy (LCE) Group, School of Chemical Engineering, Universiti Sains Malaysia, Engineering Campus, Seri Ampangan, Nibong Tebal, Pulau Pinang 143000, Malaysia

**Keywords:** Titanium dioxide, Nanoparticle, Graphene, Composite, Photocatalyst, Carbon dioxide, Methane

## Abstract

Photocatalytic reduction of carbon dioxide (CO_2_) into hydrocarbon fuels such as methane is an attractive strategy for simultaneously harvesting solar energy and capturing this major greenhouse gas. Incessant research interest has been devoted to preparing graphene-based semiconductor nanocomposites as photocatalysts for a variety of applications. In this work, reduced graphene oxide (rGO)-TiO_2_ hybrid nanocrystals were fabricated through a novel and simple solvothermal synthetic route. Anatase TiO_2_ particles with an average diameter of 12 nm were uniformly dispersed on the rGO sheet. Slow hydrolysis reaction was successfully attained through the use of ethylene glycol and acetic acid mixed solvents coupled with an additional cooling step. The prepared rGO-TiO_2_ nanocomposites exhibited superior photocatalytic activity (0.135 μmol g_cat_^−1^ h^−1^) in the reduction of CO_2_ over graphite oxide and pure anatase. The intimate contact between TiO_2_ and rGO was proposed to accelerate the transfer of photogenerated electrons on TiO_2_ to rGO, leading to an effective charge anti-recombination and thus enhancing the photocatalytic activity. Furthermore, our photocatalysts were found to be active even under the irradiation of low-power energy-saving light bulbs, which renders the entire process economically and practically feasible.

## Background

Global warming caused by large-scale emission of carbon dioxide (CO_2_) in the atmosphere and the depletion of fossil fuels are two critical issues to be addressed in the near future [1]. Great effort has been made to reduce CO_2_ emissions. Technologies involving carbon capture and geological sequestration have accelerated in the past decade [2]. Unfortunately, most of the associated processes require extraneous energy input, which may result in the net growth of CO_2_ emission. Furthermore, there are many uncertainties with the long-term underground storage of CO_2_. In this regard, the photocatalytic reduction of CO_2_ to produce hydrocarbon fuels such as methane (CH_4_) is deemed as an attractive and viable approach in reducing CO_2_ emissions and resolving the energy crisis [3,4]. Many types of semiconductor photocatalysts, such as TiO_2_[5], ZrO_2_[6], CdS [7], and combinations thereof [8] have been widely studied for this purpose.

By far the most researched photocatalytic material is anatase TiO_2_ because of its long-term thermodynamic stability, strong oxidizing power, low cost, and relative nontoxicity [9,10]. However, the rapid recombination of electrons and holes is one of the main reasons for the low photocatalytic efficiency of TiO_2_. Moreover, its wide band gap of 3.2 eV confines its application to the ultraviolet (UV) region, which makes up only a small fraction (≈5%) of the total solar spectrum reaching the earth's surface [11]. In order to utilize irradiation from sunlight or from artificial room light sources, the development of visible-light-active TiO_2_ is necessary.

In the past few years, carbon-based TiO_2_ photocatalysts have attracted cosmic interest for improved photocatalytic performance [12,13]. Graphene, in particular, has been regarded as an extremely attractive component for the preparation of composite materials [14,15]. In addition to its large theoretical specific surface area, graphene has an extensive two-dimensional *π*-*π* conjugation structure, which endows it with excellent conductivity of electrons [16]. Carriers in pristine graphene sheets have been reported to behave as massless Dirac fermions [17]. When combining TiO_2_ nanocrystals with graphene, excited electrons of TiO_2_ could transfer from the conduction band (CB) to graphene via a percolation mechanism [18]. The heterojunction formed at the interface (termed Schottky barrier) separates the photoinduced electron–hole pairs, thus suppressing charge recombination [16]. The enhancement of photocatalytic activity of graphene-based semiconductor–metal composites was first demonstrated by Kamat and co-workers in 2010 [18]. Following that, Zhang et al. [19], Shen et al. [20], and Zhou et al. [21] carried out one-step hydrothermal methods to prepare graphene-TiO_2_ hybrid materials and showed that the composites exhibited enhanced photoactivity towards organic degradation over bare TiO_2_. Fan et al. [22] fabricated P25-graphene composites by three different preparation methods, i.e., UV-assisted photocatalytic reduction, hydrazine reduction, and hydrothermal method, all of which possessed significantly improved photocatalytic performance for H_2_ evolution from methanol aqueous solution as compared to pure P25. To the best of our knowledge, the study on the use of graphene-TiO_2_ composites on the photoreduction of CO_2_ is still in its infancy. This leads to our great interest in studying the role of graphene in the composite towards the photoreduction of CO_2_ into CH_4_ gas under visible light irradiation.

In this paper, we present a simple solvothermal method to prepare reduced graphene oxide-TiO_2_ (rGO-TiO_2_) composites using graphene oxide (GO) and tetrabutyl titanate as starting materials. During the reaction, the deoxygenation of GO and the deposition of TiO_2_ nanoparticles on rGO occurred simultaneously. The photoactivity of the as-prepared rGO-TiO_2_ composite was studied by evaluating its performance in the photoreduction of CO_2_ under visible light illumination. In contrast to the most commonly employed high-power halogen and xenon lamps, we used 15-W energy-saving light bulbs to irradiate the photocatalyst under ambient condition. This renders the entire process practically feasible and economically viable. The rGO-TiO_2_ composite was shown to exhibit excellent photocatalytic activity as compared to graphite oxide and pure anatase.

## Methods

### Materials

Graphite powder, tetrabutyl titanate (TBT), acetic acid (HAc), and ethylene glycol (EG) were supplied by Sigma-Aldrich (St. Louis, MO, USA). All reagents were of analytical reagent grade and were used without further purification.

### Synthesis of reduced graphene oxide-TiO_2_ composite

Graphite oxide was prepared from graphite powder by modified Hummers' method [23-25]. The detailed experimental procedure is given in Additional file 1. To obtain GO sheets, graphite oxide was dispersed into distilled water (0.5 g L^−1^) and ultrasonicated for 1 h at ambient condition. The solution was then chilled to ≈ 5°C in an ice bath. Meanwhile, a titanium precursor composed of 1.5 mL TBT, 7.21 mL EG, and 1.14 mL HAc was also chilled to ≈ 5°C in an ice bath. The mixture was then added dropwise into the chilled GO aqueous solution under vigorous stirring. Subsequently, the GO-TiO_2_ stock solution was transferred into a 200-mL Teflon-lined stainless steel autoclave and was heated at 180°C for 8 h. The greyish-black precipitate was harvested by centrifugation (5,000 rpm, 30 min) and was washed with ethanol several times to remove undecorated TiO_2_ particles, unreacted chemicals, and residual EG. Finally, the product was dried in an air oven at 60°C overnight before characterization.

### Characterization

Morphology observation was performed using an SU-8010 field emission scanning electron microscope (FESEM; Hitachi Ltd., Tokyo, Japan) equipped with an Oxford-Horiba Inca XMax50 energy-dispersive X-ray (EDX; Oxford Instruments Analytical, High Wycombe, England). High-resolution transmission electron microscopy (HRTEM) was conducted with a JEOL JEM-2100 F microscope (JEOL, Tokyo, Japan) operating at 200 kV. The X-ray powder diffraction data were obtained on a Bruker AXS (Madison, WI, USA) D8 Advance X-ray diffractometer with CuKα radiation (*λ* = 0.15406 nm) at a scan rate (2*θ*) of 0.02° s^−1^. The accelerating voltage and applied current were 40 kV and 40 mA, respectively. The crystallite size measurements of anatase TiO_2_ were quantitatively calculated using Scherrer's equation (*d* = *kλ*/*β* cos *θ*) where *d* is the crystallite size, *k* is a constant (=0.9 assuming that the particles are spherical), *β* is the full width at half maximum (FWHM) intensity of the (101) peak in radians, and *θ* is Bragg's diffraction angle [26]. Raman spectra were recorded at room temperature on a Renishaw inVia Raman microscope (Renishaw, Gloucestershire, UK). UV-visible absorption spectra for the samples were collected with an Agilent Cary-100 UV-visible spectroscope (Agilent Technologies, Santa Clara, CA, USA). A Nicolet iS10 Fourier transform infrared (FTIR) spectrometer (Thermo Scientific, Logan, UT, USA) was used to record the FTIR spectra of all samples.

### Photocatalytic CO_2_ reduction experiment

The photocatalytic experiment for the reduction of CO_2_ was conducted at ambient condition in a homemade, continuous gas flow reactor. A 15-W energy-saving daylight bulb (Philips, Amsterdam, Netherlands) was used as the visible light source. The catalyst powder was first fixed into a quartz reactor. Highly pure CO_2_ (99.99%) was bubbled through water (sacrificial reagent) to introduce a mixture of CO_2_ and water vapor into the photoreactor at ambient pressure. Prior to irradiation, CO_2_ was purged inside the reactor for 30 min to remove the oxygen and to ensure complete adsorption of gas molecules. The light source was then turned on to initiate photocatalytic reaction. The generated gases were collected at 1-h intervals and were analyzed by a gas chromatograph (GC), equipped with a flame ionization detector (FID) (Agilent, 7890A) to determine the yield of CH_4_. Control experiments were also carried out in the dark, and no product gases were detected for all tested catalysts. This indicates that light irradiation was indispensable for the photoreduction of CO_2_ to CH_4_.

## Results and discussion

### Synthesis strategy

**Figure 1:**
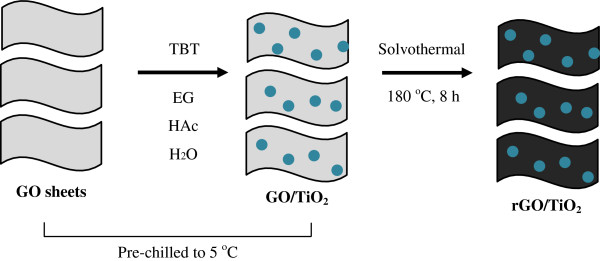
**Procedure for the synthesis of rGO-TiO**_**2 **_**nanocomposites.**

GO sheets possess a rich assortment of oxygen-containing groups, such as epoxide, hydroxyl, carbonyl, and carboxylic groups [27]. Therefore, titanium alkoxides, in this case TBT, can be readily grafted onto the surface of GO through chemical adsorption at the molecular level [28]. On the other hand, it is widely known that titanium alkoxides are extremely sensitive to water. Rapid decomposition of the titanium precursor would result in the agglomeration of TiO_2_ crystals as well as hinder the homogeneous growth of TiO_2_ onto GO. Hence, EG and HAc were introduced into the mixture to co-control the hydrolysis rate of TBT [29]. In addition, the mixtures were also prechilled in an ice bath to further reduce the hydrolysis rate. During the solvothermal treatment, GO was reduced to rGO, and TiO_2_ nanoparticles formed on the rGO surface simultaneously. The preparation strategy is illustrated in Figure 1.

### Characterization of reduced graphene oxide-TiO_2_ composites

**Figure 2:**
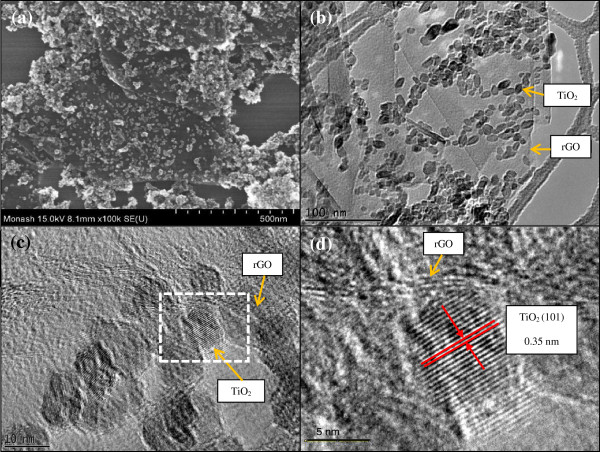
**Electron microscopy of the rGO-TiO**_**2 **_**composites. (a)** FESEM image, **(b, c)** HRTEM images, and **(d)** enlarged HRTEM image of a selected rGO-TiO_2_ heterojunction.

The surface morphology and structure of the rGO-TiO_2_ nanocomposite were characterized using FESEM and HRTEM. From Figure 2a, b, it is observed that the surface of rGO sheets was packed densely with TiO_2_ nanoparticles, which displayed a good combination of rGO and TiO_2_. Despite that, the profile of a single TiO_2_ nanoparticle could be clearly distinguished, indicating that the aggregation of TiO_2_ was well prevented during the preparation process. The TiO_2_ particles were also found to exhibit a narrow size distribution with an average crystallite size of 12 nm. The corresponding HRTEM images (Figure 2c, d) clearly showed the lattice fringes of rGO, which were parallel to the edges of the TiO_2_ nanoparticles. The lattice spacing of TiO_2_ was measured to be *ca.* 0.35 nm, which corresponds to the (101) plane of anatase TiO_2_ (JCPDS no. 2101272). The rGO sheets were composed of a mixture of two to five layers based on HRTEM observations.

It is known that few-layer rGO sheets have the tendency to aggregate back to the graphite structure due to strong van der Waals interaction [30]. Therefore, the crystallization of TiO_2_ on the surface of rGO was particularly helpful in overcoming this interaction, which could ultimately alleviate the agglomeration and restacking of the graphene sheets. In addition, the intimate connection would allow the electrons to transfer easily from TiO_2_ to rGO sheets during the photoexcitation process, which could significantly increase the separation of photoinduced charges and enhance the photocatalytic activity.

**Figure 3:**
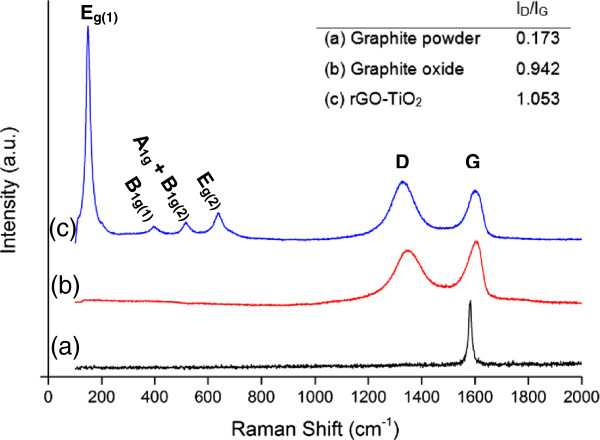
**Raman spectra of (spectrum a) graphite powder, (spectrum b) graphite oxide, and (spectrum c) rGO-TiO**_**2 **_**composite.**

Raman spectroscopy has been accepted to be a powerful and nondestructive tool to characterize the quality of graphitic materials. The significant structural changes resulting from the solvothermal reaction process from GO to rGO were also reflected in the Raman spectra. Figure 3 (spectra a and b) shows the Raman measurements of graphite before and after the modified Hummers' method. There were two characteristic peaks in the spectrum of graphite: the D (disordered) peak centered at 1,347 cm^−1^ and the G (graphitic) peak at 1,582 cm^−1^. The D band is attributed to the disruption of the symmetrical hexagonal graphitic lattice as a result of edge defects, internal structural defects, and dangling bonds. On the other hand, the G band is due to the in-plane stretching motion of symmetric *sp*^2^ C-C bonds. A narrower G band indicates that fewer functional groups (i.e., non-C-C bonds) are present [31]. After the oxidation of graphite, the Raman spectrum of graphite oxide showed that the G band was broadened, while the intensity of the D band was increased significantly. These observations were ascribed to the substantial decrease in size of the in-plane *sp*^2^ domains, resulting from the introduction of oxygen-containing groups. In addition, the shift in the G band from 1,582 to 1,609 cm^−1^ was possibly due to the presence of isolated double bonds on the carbon network of graphite oxide [32]. It has been reported that isolated double bonds tend to resonate at higher frequencies as compared to the G band of graphite [33]. Figure 3 (spectrum c) shows the Raman spectrum of the rGO-TiO_2_ composite. The typical modes of anatase could be clearly observed, i.e., the E_g(1)_ peak (148 cm^−1^), B_1g(1)_ peak (394 cm^−1^), E_g(2)_ peak (637 cm^−1^), and the A_1g_ + B_1g(2)_ modes centered at 512 cm^−1^, respectively [34]. The two characteristic peaks at about 1,328 and 1,602 cm^−1^ for the graphitized structures were also observed in the Raman spectrum of the rGO-TiO_2_ composite. The composite showed an increase in *I*_D_/*I*_G_ ratio as compared to graphite oxide, indicating a decrease in the average size of the in-plane *sp*^2^ domains of C atoms in the composite, which is similar to that observed in chemically reduced GO [35].

**Figure 4:**
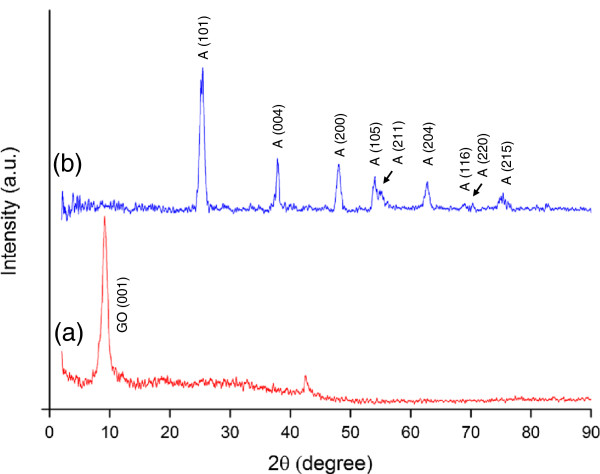
**XRD spectra of (spectrum a) graphite oxide and (spectrum b) rGO-TiO**_**2 **_**composite.**

Figure 4 shows the XRD patterns of graphite oxide and the rGO-TiO_2_ composite. The XRD pattern of graphite oxide (Figure 4, spectrum a) showed that the interlayer distance obtained from the characteristic (001) peak is ≈ 0.93 nm (2*θ* = 9.50°), which matches well with the values reported in literature [16,20,36]. This confirmed that most of the graphite powder was oxidized into graphite oxide by expanding the *d* spacing from 0.34 to 0.93 nm [20,37]. The large interlayer distance of graphite oxide could be attributed to the presence of oxygen-containing functional groups such as hydroxyl, carboxyl, carbonyl, and epoxide [38]. Figure 4 (spectrum b) shows the XRD patterns of the rGO-TiO_2_ composite. The peaks at 25.3°, 37.8°, 48°, 53.9°, 55.1°, 62.7°, 68.8°, 70.3°, and 75.0° can be indexed to the (101), (004), (200), (105), (211), (204), (116), (220), and (215) crystal planes of a pure tetragonal anatase phase (space group, I4_1_/amd; JCPDS no. 21–1272) with lattice constants *a* = 3.78 Å and *c* = 9.50 Å [39,40]. Crystal facet (101) was the main crystal structure of the anatase TiO_2_ due to its maximum peak intensity. No rutile phase was detected due to the low reaction temperature employed in this work. The average crystal size of the TiO_2_ nanoparticles in the composite was calculated to be *ca.* 8.1 nm based on Scherrer's equation. No diffraction peaks from impurities and other phases could be detected, thus indicating that the product was pure and well crystallized. Notably, the typical diffraction peaks of graphene or GO were not found in the XRD pattern of the composite. A possible reason for this observation was that the most intense diffraction peak of graphene (2*θ* = 24.5°) [41] could be shielded by the main peak of anatase TiO_2_ at 25.3°.

**Figure 5:**
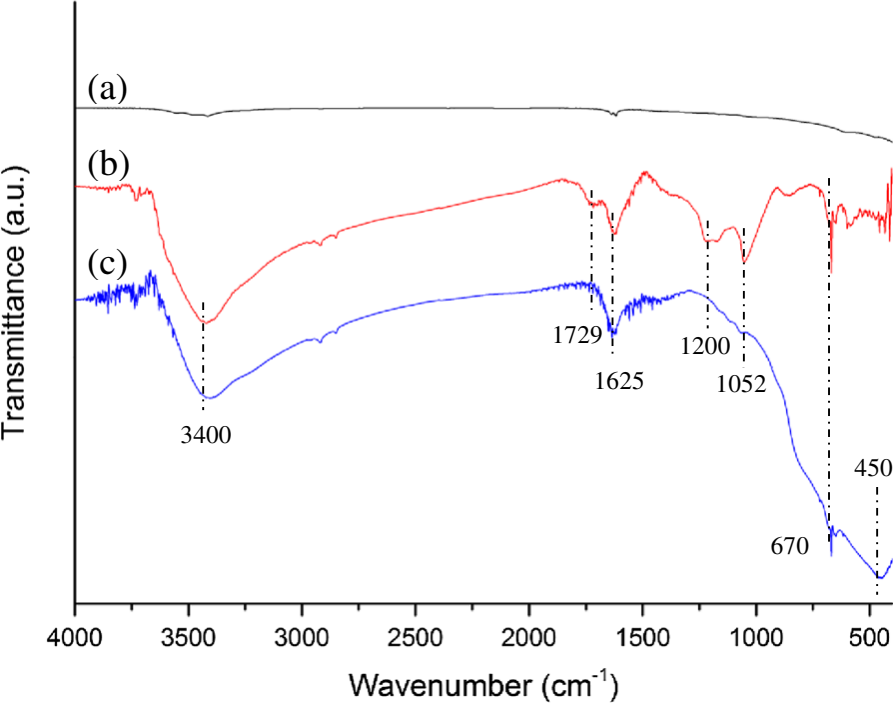
**FTIR spectra of (spectrum a) graphite powder, (spectrum b) graphite oxide, and (spectrum c) rGO-TiO**_**2 **_**composite.**

Figure 5 shows the FTIR spectra of graphite powder, graphite oxide, and the rGO-TiO_2_ composite. While no significant peaks were observed in raw graphite, graphite oxide was found to exhibit several characteristic absorption bands of oxygen-containing groups (Figure 5, spectrum b). The absorption peaks included 870 cm^−1^ for aromatic C-H deformation [42], 1,052 cm^−1^ for C-O stretching [21], 1,220 cm^−1^ for phenolic C-OH stretching [42], 1,625 cm^−1^ for the hydroxyl groups of molecular water [43], 1,729 cm^−1^ for C = O stretching [20], and a broad peak at 3,400 cm^−1^ for the O-H stretching vibrations of C-OH groups [44]. The small peaks at 2,854 and 2,921 cm^−1^ in the spectrum were attributed to the CH_2_ stretching vibration [45]. Figure 5 (spectrum c) shows the FTIR measurement for the rGO-TiO_2_ composite. It can be observed that the intensities of absorption bands of oxygen-containing functional groups such as C-O (1,052 cm^−1^) were dramatically reduced. The C-OH and carbonyl C = O bands at 1,200 and 1,729 cm^−1^, respectively, were also found to have disappeared for the rGO-TiO_2_ composite. However, it can be seen that the spectrum retains a broad absorption band centered at 3,400 cm^−1^, which was attributed to the residual O-H groups of rGO. These results implied that GO was not completely reduced to graphene through the solvothermal treatment but was instead partially reduced to rGO, which possessed residual oxygen-containing functional groups. Therefore, TiO_2_ could be susceptible to interactions with these functional groups in the nanocomposites [45]. The spectrum also showed strong absorption bands at 450 and 670 cm^−1^, indicating the presence of Ti-O-Ti bond in TiO_2_[46].

**Figure 6:**
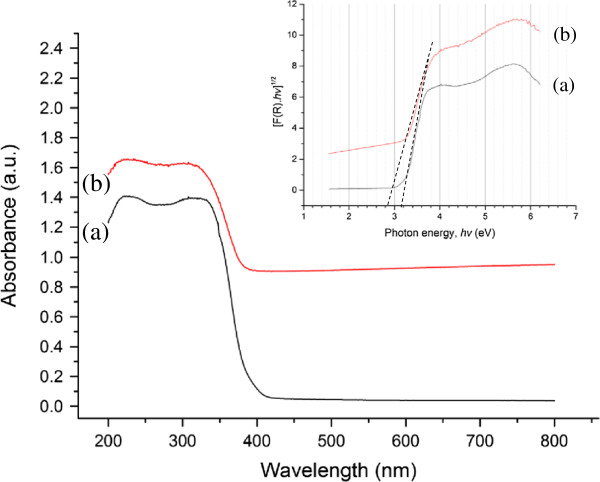
**UV–vis diffuse reflectance spectra of (spectrum a) pure anatase and (spectrum b) rGO-TiO**_**2 **_**.** Inset: plot of transformed KM function [F(R).*hv*]^1/2^ vs. *hv* for pure anatase and rGO-TiO_2_.

UV-visible (UV–vis) spectroscopy has been proven to be an effective optical characterization technique to understand the electronic structure of semiconductors. Figure 6 (spectra a and b) shows the UV–vis diffuse reflectance spectra (DRS) of pure anatase and the rGO-TiO_2_ composite, respectively. Both samples displayed a typical absorption with an intense transition in the UV region of the spectra, which was assigned to the intrinsic band gap absorption of TiO_2_ resulting from the electron transitions from the valence band to the conduction band (O_2p_ → Ti_3d_) [26]. In comparison with pure anatase, a substantial red shift to higher wavelength in the absorption edge of the rGO-TiO_2_ composite could be observed, therefore indicating a narrowing of band gap with the introduction of rGO. The optical band gaps of pure anatase and rGO-TiO_2_ were determined using a Tauc plot of the modified Kubelka-Munk (KM) function with a linear extrapolation (see inset of Figure 6). The approximated band gaps of pure anatase and rGO-TiO_2_ were 3.20 and 2.90 eV, respectively. This supported the qualitative observation of a red shift in the absorption edge of the composite as compared to pure anatase. The narrowing of band gap could be ascribed to the chemical bonding between TiO_2_ and the specific sites of carbon during the solvothermal treatment, which is analogous to the case of carbon nanotube (CNT)-TiO_2_ composite materials [47,48]. Pure anatase exhibited no absorption above its absorption edge, indicating that it was not photocatalytically responsive in the visible light region. In contrast, the introduction of rGO resulted in a continuous absorption band ranging from 400 to 800 nm, which was in agreement with the greyish-black color of the sample. The increased absorption intensity of light for the rGO-TiO_2_ composites suggested that they could exhibit an enhanced photocatalytic activity for a given reaction. This hypothesis was confirmed by its use in the photocatalytic reduction of CO_2_ under ambient condition.

### Photocatalytic reduction of CO_2_ with H_2_O and mechanism

**Figure 7:**
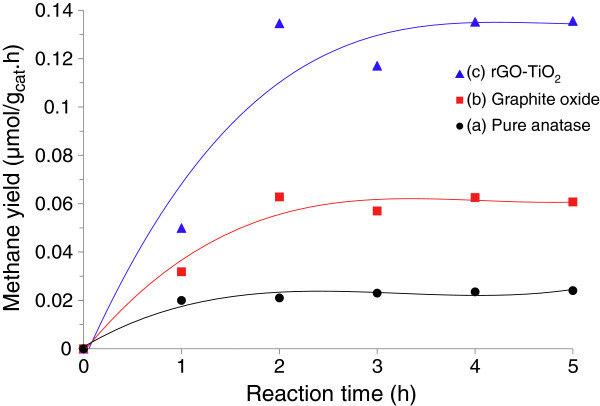
**Time dependence on the photocatalytic formation rate of CH**_**4**_**.** Over **(**curve **a)** pure anatase, **(**curve **b)** graphite oxide, and **(**curve **c)** rGO-TiO_2_ under visible light irradiation.

The photocatalytic performance of our rGO-TiO_2_ nanocomposite was measured by the photoreduction of CO_2_ under visible light irradiation using water vapor as a scavenger. Graphite oxide and pure anatase were separately tested under similar conditions. Control experiments indicated that no appreciable CH_4_ formation was detected in the absence of either light irradiation or photocatalyst, confirming that CH_4_ gas was produced by photocatalytic reactions. According to the procedure described in the ‘Methods’ section, the yield of CH_4_ gas (μmol g_cat_^−1^ h^−1^) was calculated and plotted in Figure 7 as a function of reaction time (h). The photocatalytic activity of CO_2_ reduction was found to follow the order rGO-TiO_2_ < graphite oxide < TiO_2_. Pure anatase TiO_2_ exhibited the lowest photocatalytic performance due to its limited photoresponse range under visible light irradiation. Graphite oxide showed an improvement in performance where it reached a maximum yield of 0.0628 μmol g_cat_^−1^ h^−1^ before leveling off after 2 h of testing. Yeh et al. [49] have demonstrated the use of graphite oxide as a photocatalyst for the steady evolution of H_2_ from water splitting. To the best of our knowledge, no paper has reported the use of graphite oxide in the conversion of CO_2_ into CH_4_ gas. This finding is interesting as it highlights the possibility of using inexpensive and abundant graphitic materials as photocatalysts to convert CO_2_ under solar illumination. Graphite oxide is the intermediate state between graphite and graphene [27]. It has been shown that its band gap is dependent on the number of oxygenated sites [49]. Also, the isolated *sp*^2^ clusters on graphite oxide with oxygen-containing functional groups such as C-OH and C-O-C would lead to the localization of electron–hole pairs on its basal plane [49,50]. These photoinduced charges would then migrate to the surface of graphite oxide and act as oxidizing and reducing sites, respectively, to react with the adsorbed reactants (in this case, CO_2_ and H_2_O vapor). Among all three samples, the rGO-TiO_2_ nanocomposite exhibited the highest photocatalytic performance towards CO_2_ reduction. The maximum CH_4_ product yield of 0.135 μmol g_cat_^−1^ h^−1^ was attained after 4 h of reaction. A slight decrease in yield can be observed at the third hour of reaction. This deviation is not uncommon in continuous gas-phase photocatalytic systems, and similar trends have been reported in literature [51,52]. The rGO-TiO_2_ nanocomposite was shown to exhibit an enhancement factor of 2.1 and 5.6 as compared to graphite oxide and pure anatase, respectively. It is interesting to note that the rGO-TiO_2_ composite was active even under the irradiation of low-power, energy-saving light bulbs. The use of high-intensity halogen and xenon arc lamps was not required for the photoexcitation process to take place.

On the basis of our experimental data, it is proposed that the synergistic dyade structure of the rGO-TiO_2_ composite provided access to an optically active charge transfer transition. In other words, rGO and anatase TiO_2_ formed a joint electronic system. The enhancement in photocatalytic activity could be attributed to the combined effect of several concomitant factors. Firstly, the band gap narrowing of the rGO-TiO_2_ composite (3.2 eV → 2.90 eV) allowed an enhanced absorption of visible light. The CB of anatase TiO_2_ and the work function of rGO are −4.2 eV [53] and −4.42 eV [46], respectively. Such energy levels were beneficial for the photogenerated electrons to transfer from the TiO_2_ CB to the rGO, which could effectively separate the charge carriers and hinder electron–hole recombination. In the absence of rGO, most of these charges tend to recombine rapidly without undergoing any chemical reaction [30]. This is primarily due to the adsorption kinetic of the CO_2_ molecules (10^−8^ to 10^−3^ s) on TiO_2_ being slower than the electron–hole recombination time (10^−9^ s) [47,54].

**Figure 8:**
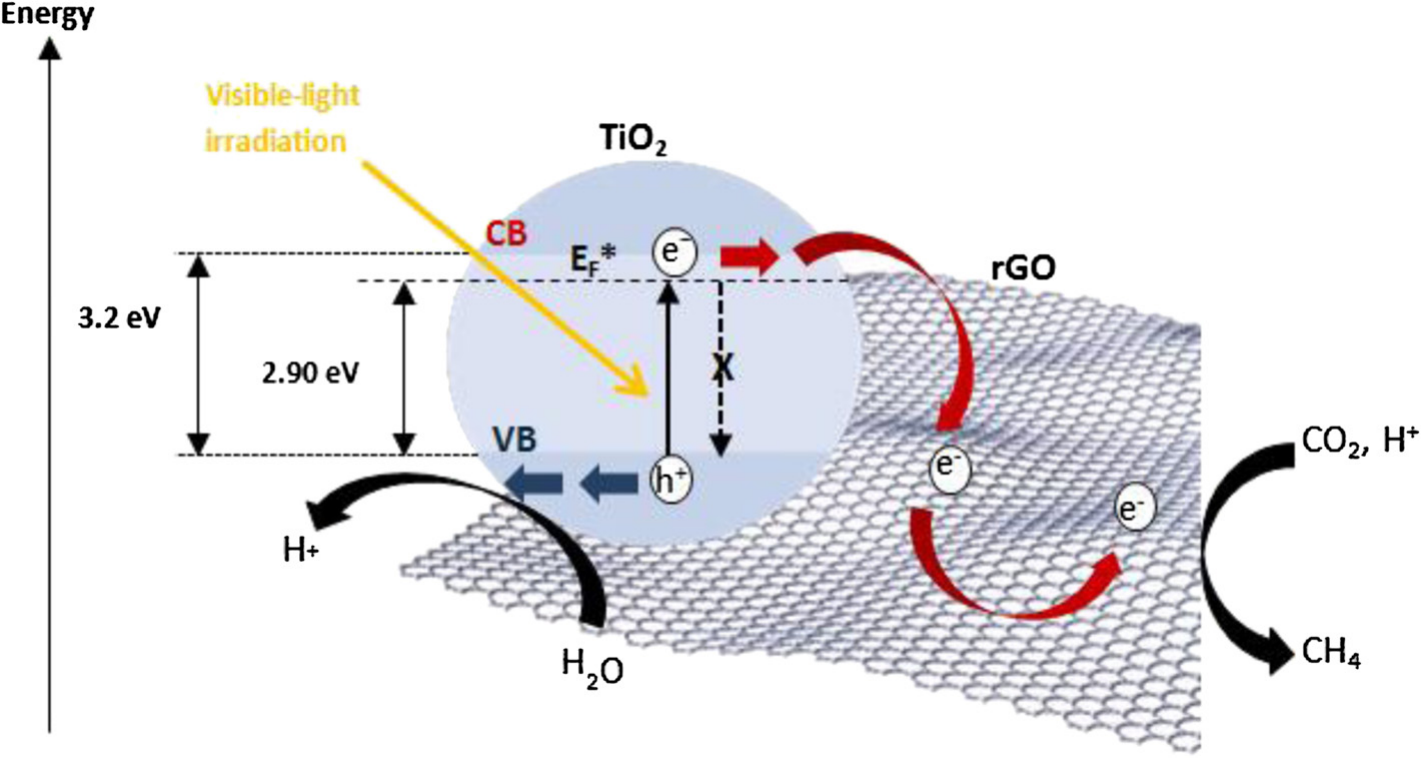
**Charge transfer and separation in the rGO-TiO**_**2**_**composite.** Schematic illustrating the charge transfer and separation in the rGO-TiO_2_ composite for the photoreduction of CO_2_ under visible light irradiation with the introduction of a new energy level, *E*_F_^*^.

In addition, the two-dimensional and planar *π*-conjugation structure of rGO endowed it with excellent conductivity of electron [16,55]. As we know, one photon can usually induce the transfer of only one electron in photochemical reactions. However, the photocatalytic reduction of CO_2_ required a multi-electron process to yield CH_4_.Therefore, in the rGO-TiO_2_ composite, rGO served as an electron collector and transporter to effectively separate the photogenerated electron–hole pairs. This in turn lengthened the lifetime of the charge carriers, which could be advantageous for overcoming this obstacle to improve the selective formation of CH_4_ gas. During the photocatalytic reaction, a large number of electrons would be produced due to the highly dispersed TiO_2_ nanoparticles over the rGO sheets (see Figure 2a,b). Furthermore, the large specific surface area of rGO also increased the adsorption of the CO_2_ molecules, thus favoring the formation of CH_4_. The mechanisms of photocatalytic enhancement over the rGO-TiO_2_ composite are depicted in Figure 8.

The photocatalytic conversion of CO_2_ to CH_4_ over the rGO-TiO_2_ composite can be understood using the energy band theory, which is based on the relative positions of CB, VB, and oxidation potentials. In general, the overall mechanism of the CO_2_ transformation process is a sequential combination of H_2_O oxidation and CO_2_ reduction. In the rGO-TiO_2_ composite, the TiO_2_ nanoparticles exhibited an intimate contact with the rGO sheet. The *d* orbital (CB) of TiO_2_ and the *π* orbital of rGO matched well in energy levels, thus resulting in a chemical bond interaction to form *d*-*π* electron orbital overlap [56]. The CB flatband potential of TiO_2_ is −0.5 V (vs. normal hydrogen electrode (NHE), pH = 7) [57], which is more negative than the reduction potential of CO_2_/CH_4_ (−0.24 V vs. NHE, pH = 7) [58] acts as a donor. This indicated that the photogenerated electrons and holes on the irradiated rGO-TiO_2_ composites can react with adsorbed CO_2_ and H_2_O to produce CH_4_ via an eight-electron reaction. The major reaction steps in the photocatalytic CO_2_ reduction process can be summarized by Equations 1, 2 and 3

(1) $$\mathrm{Graphene}‒{\mathrm{TiO}}_{2}\overset{\mathrm{hv}}{\to }\;\mathrm{graphene}\left(e^{‒}\right)/{\mathrm{TiO}}_{2}\left(h_{\mathrm{VB}}^{+}\right)$$

(2) $${\mathrm{TiO}}_{2}\;\left(2h_{\mathrm{VB}}^{+}\right)+H_{2}O\to 2H^{+}+\frac{1}{2}O_{2}\;\left[E^{o}=+0.82\;V\right]$$

(3) $${\mathrm{CO}}_{2}+8H^{+}+8e^{‒}\to {\mathrm{CH}}_{4}+2H_{2}O\;\left[E^{o}=-0.24\;V\right]$$

## Conclusions

In summary, a visible-light-active rGO-based TiO_2_ photocatalyst was developed by a facile, one-pot solvothermal method. To control the hydrolysis reaction rate of water-sensitive TBT, we employed EG and HAc mixed solvent coupled with an additional cooling step in our synthesis procedure. Anatase TiO_2_ nanoparticles with an average crystallite size of 12 nm were homogeneously anchored onto the rGO sheets with close interfacial contact. The activity of the rGO-TiO_2_ composite was tested by the photocatalytic reduction of CO_2_ under visible light irradiation. The composite displayed excellent photocatalytic activity, achieving a maximum CH_4_ product yield of 0.135 μmol g_cat_^−1^ h^−1^, which is 2.1- and 5.6-fold higher than that achieved by graphite oxide and pure anatase. The incorporation of rGO into the composite led to the reduction of band gap, rendering the rGO-TiO_2_ hybrid material sensitive to visible light irradiation (λ < 400 nm). In addition, the photoinduced electrons can easily migrate to the rGO moiety, leading to the efficient separation and prolonged recombination time of charge carriers. These contributions, together with increased reactant adsorption, are the primary factors in the enhancement of the rGO-TiO_2_ photoactivity. In contrast to the most commonly used high-power halogen and xenon arc lamps, we demonstrated that our photocatalysts were active even under the irradiation of low-power, energy-saving light bulbs. Interestingly, we have also found that graphite oxide was active in the photoconversion of CO_2_ into CH_4_ gas under visible light irradiation. Ongoing research is being carried out to develop more complex rGO-based semiconducting materials for the efficient conversion of CO_2_. We believe that our findings could open up a scalable and cost-effective approach to obtain robust materials for photocatalytic applications.

## Competing interests

The authors declare that they have no competing interests.

## Authors’ contributions

LLT and WJO conceived and designed the experimental strategy. LLT performed the experiments and prepared the manuscript. SPC and ARM supervised the whole work and revised the manuscript. All authors read and approved the final manuscript.

## Supplementary Material

Additional file 1 **Preparation of graphite oxide powder.** Detailed experimental procedure with two accompanying figures. Click here for file
