# Supplementary material for: Reduced graphene oxide-TiO2 nanocomposite as a promising visible-light-active photocatalyst for the conversion of carbon dioxide
Source: Nanoscale Res Lett. 2013 Nov 6;8(1):465. doi: 10.1186/1556-276X-8-465 (PMC3827867; doi:10.1186/1556-276X-8-465)
Supplement: Additional file 1 — Preparation of graphite oxide powder. Detailed experimental procedure with two accompanying figures. [file 1556-276X-8-465-S1.pdf]

Additional File 1

Reduced graphene oxide-TiO_2 nanocomposite as a promising visible-light-active photocatalyst for the conversion of carbon dioxide

Lling-Lling Tan,[a] Wee-Jun Ong,[a] Siang-Piao Chai[*a] and Abdul Rahman Mohamed[b]

[a] *Low Carbon Economy (LCE) Group, Chemical Engineering Discipline, School of Engineering, Monash University, Jalan Lagoon Selatan, Bandar Sunway, 46150, Selangor, Malaysia.*

[b] *Low Carbon Economy (LCE) Group, School of Chemical Engineering, Universiti Sains Malaysia, Engineering Campus, Seri Ampangan, 14300 Nibong Tebal, Pulau Pinang, Malaysia.*

* Corresponding author.

Tel: +603-5514 6234; Fax: +603-5514 6207

E-mail address: chai.siang.piao@monash.edu

1. Preparation of graphite oxide powder

Graphite oxide was prepared using graphite powder as the starting material *via* the Modified Hummers' Method. In detail, 3 g of graphite powder (Sigma Aldrich, < 45micron, > 99.99%) was added into an 80 ^{o}C mixture containing 12 mL of concentrated H_2SO_4 (Chemolab supplies, 95-97%), 2.5 g of P_2O_5 (Sigma Aldrich, ≥ 98.0%) and 2.5 g of $K_2S_2O_8$ (Sigma Aldrich, ≥ 99.0%). The mixture was stirred for 4.5 h with the temperature maintaining at 80 ^{o}C. Next, the mixture was cooled to room temperature and was then diluted with 0.5 L of deionized (DI) water. Successively, the mixture was filtered over a Nylon membrane with 0.2 μm pore size and washed with DI water until the pH of the filtrate water became neutral. The product was then dried in air overnight. This pre-oxidized graphite was then dispersed into 120 mL of cold (0 ^{o}C) concentrated H_2SO_4. Then, 15 g of $KMnO_4$ (Sigma Aldrich, ≥ 99.0%) was gradually added under stirring and the temperature of the mixture was kept below 20 ^{o}C by cooling. This step resulted in the formation of a thick dark green paste. Subsequently, the mixture was stirred at room temperature for 2 h, and then slowly diluted with 250 mL of DI water in an ice bath to keep the temperature below 50 ^{o}C (Note: The DI water was added in 5 mL aliquots to avoid overflow of mixture due to rapid temperature rise with foaming by water addition). After another 2 h of stirring, the dark brownish solution was further diluted with 700 mL of DI water. 20 mL of H_2O_2 (R&M Chemicals, 30%) was then slowly added into the mixture and a brilliant yellow product was formed along with bubbling. The mixture was filtered and washed with 1 L of HCl (Merck, 37% diluted to 10%) to remove metal ions followed by 1 L of DI water to remove the acid. Successively, the filter cake was dispersed in water by mechanical agitation. Low-speed centrifugation was done at 1000 rpm for 2 min. The supernatant then underwent high-speed centrifugation steps at 8000 rpm for 30 min to remove small pieces of GO and water soluble by-products. Finally, the sediment was dried in air at 60 ^{o}C for 24 h. The product (graphite oxide) was stored in powder form.

2. Figures

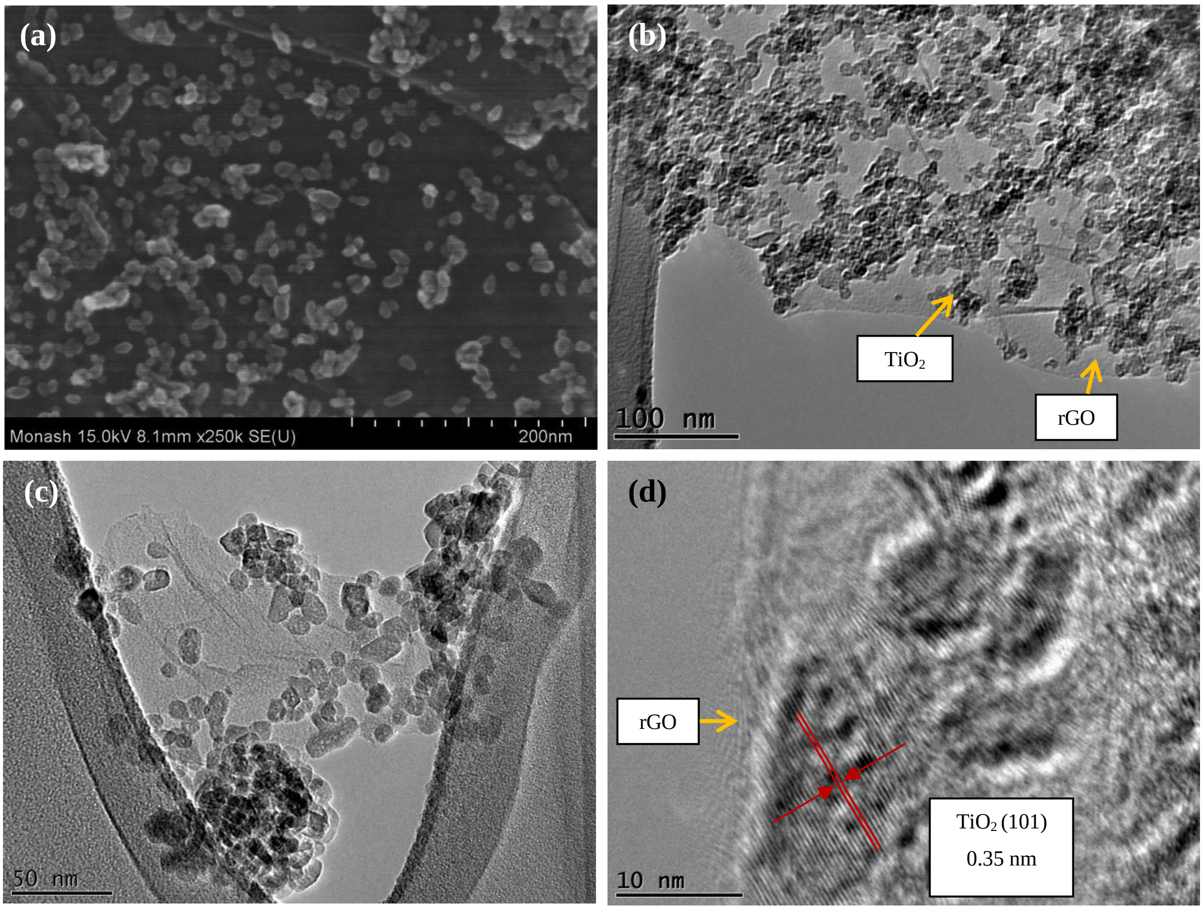

Figure S1 (a) FESEM and (b-d) HRTEM images of the rGO-TiO_2 composite

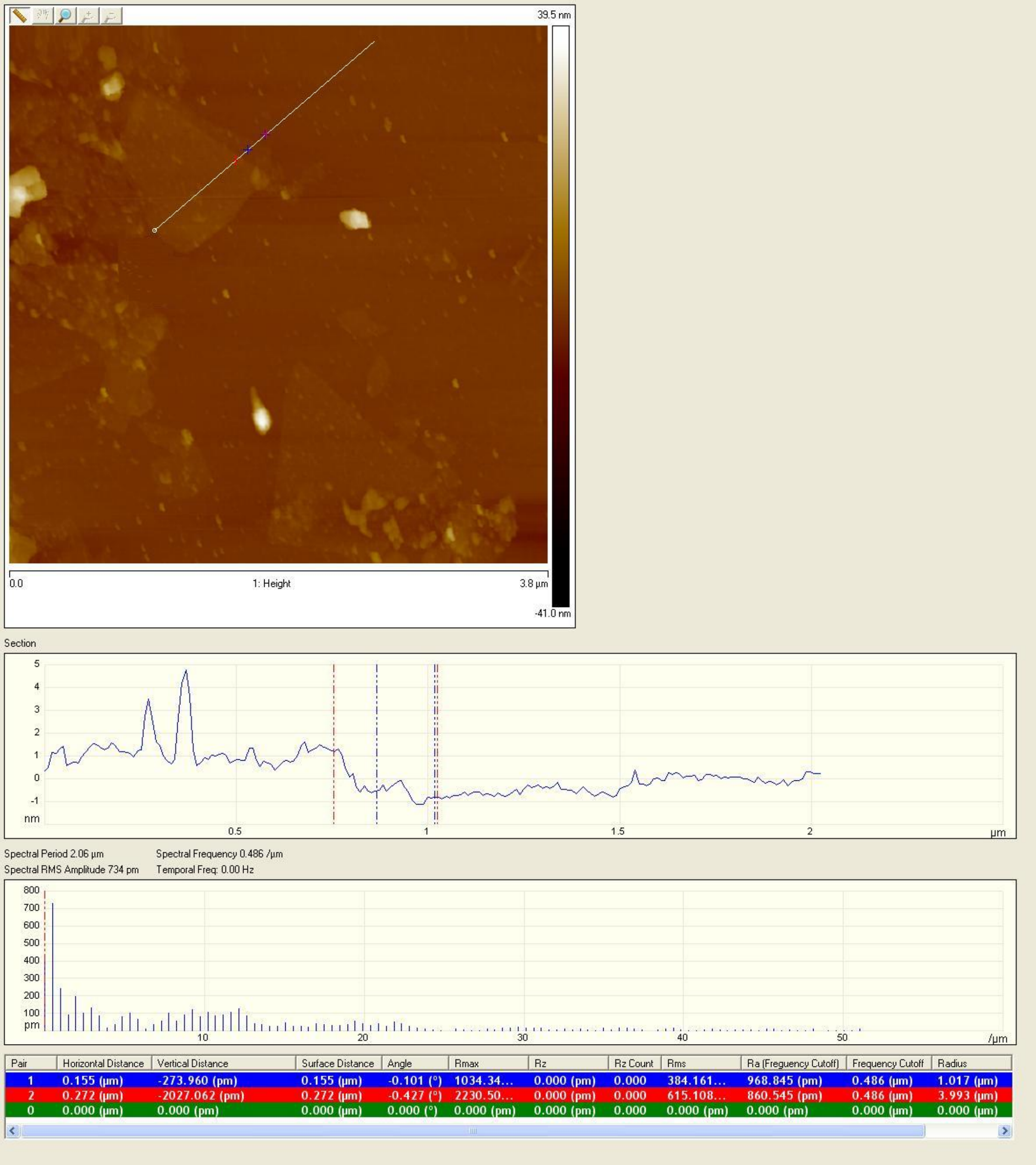

Figure S2 Tapping-mode AFM image of GO and the corresponding height profiles along the lines shown in GO image. While a pristine graphene sheet is atomically flat with a well-known thickness of 0.34 nm [1], GO is expected to be thicker due to the presence of bulky carboxyl, carbonyl and epoxide groups above and below the GO plane. Single-sheet GO is normally found to be in the range of 0.9 – 1.3 nm when analyzed through AFM [2]. Therefore, results suggest that the graphite oxide was exfoliated down to single and bi-layer GO sheets.

References

1. Geng J, Kong B-S, Yang SB, Jung H-T: **Preparation of graphene relying on porphyrin exfoliation of graphite**. *Chem Commun* 2010, **46**(28):5091-5093.
2. Stankovich S, Dikin DA, Piner RD, Kohlhaas KA, Kleinhammes A, Jia Y, Wu Y, Nguyen ST, Ruoff RS: **Synthesis of graphene-based nanosheets via chemical reduction of exfoliated graphite oxide**. *Carbon* 2007, **45**(7):1558-1565.
